# Vitamin D supplementation and falls in residential aged care: A longitudinal multisite cohort study

**DOI:** 10.1016/j.bonr.2024.101791

**Published:** 2024-07-23

**Authors:** Nasir Wabe, Isabelle Meulenbroeks, Desiree C. Firempong, Magdalena Z. Raban, Amy D. Nguyen, Jacqueline T. Close, Stephen R. Lord, Johanna I. Westbrook

**Affiliations:** a Centre for Health Systems and Safety Research, Australian Institute of Health Innovation, Macquarie University, Australia; b St Vincent's Clinical School, University of New South Wales Medicine, UNSW Sydney, Sydney, Australia; c Neuroscience Research Australia, UNSW Sydney, Sydney, Australia; d Prince of Wales Clinical School, University of New South Wales, Sydney, Australia; e School of Population Health, UNSW, Sydney, Australia

**Keywords:** Vitamin D, Falls, Aged care, RAC, Older people

## Abstract

**Background:**

Vitamin D is vital for musculoskeletal health, and supplementation may lower risk of falls. Past research in residential aged care (RAC) settings on the effects of vitamin D on falls have reported inconclusive findings, partly due to study design limitations. We utilised a longitudinal study design to assess the association between the use of vitamin D and falls over 36 months in RAC.

**Method:**

A longitudinal cohort study was conducted using routinely collected electronic data spanning 9 years from 27 RAC facilities in Sydney, New South Wales, Australia. The study included 4520 permanent residents aged 65 years or older who were admitted for the first time from 1 July 2014 and stayed for a minimum of one month. We identified daily vitamin D usage over 36 months, and measured adherence using the Proportion of Days Covered (PDC) metric. A PDC value of ≥80 % signifies optimal adherence. Primary outcomes were the number of all falls and injurious falls. A rolling time-varying predictor-outcome approach and Generalized Estimating Equations (GEE) were applied to determine the longitudinal link between vitamin D supplement use and subsequent risk of falls.

**Results:**

Over two-thirds of residents (67.8 %; *n* = 3063) received vitamin D supplements during their stay, with a median PDC of 74.8 % among users, and 44.6 % (*n* = 1365) achieving optimal adherence. Increasing age, osteoporosis or fracture history, and dementia were associated with a greater likelihood of achieving optimal adherence. Crude fall incident rates were 8.05 and 2.92 incidents per 1000 resident days for all falls and injurious falls respectively. After accounting for relevant demographic and clinical factors, no significant links were observed between vitamin D supplement usage and fall outcomes: all falls (Incident Rate Ratio [IRR] 1.01; 95 % CI 1.00–1.02; *P* = 0.237) and injurious falls (IRR 1.01; 95 % CI 1.00–1.02; *P* = 0.091).

**Conclusion:**

Vitamin D supplementation was not associated with a reduced risk of falls, suggesting it is not an effective intervention for preventing falls in RAC. While clinicians should ensure adequate vitamin D intake for residents' nutritional and bone health, it should not be a standalone falls prevention intervention in RAC populations.

## 1 Introduction

Falls in older adults cause substantial burden, including injuries, heightened care needs, and even premature death. Falls-associated treatment costs in Australia are estimated at $4.3 billion and were the fourth highest area of healthcare spending between 2018 and 2019 (Australian Institute of Health and Welfare, n.d.). Older adults in residential aged care (RAC), also known as nursing homes and long-term care facilities, have a higher risk of falls compared to their counterparts in the community (World Health Organization, 2008), mainly due to increased age-related comorbidities as well as physical and cognitive functional impairments. As the number of older adults entering RAC increases (Australian Bureau of Statistics, n.d.), it is critical that effective falls prevention interventions are implemented in this setting to improve care quality and safety and minimise economic burden in this high risk population (Gustavo et al., 2010).

Vitamin D plays an essential role in supporting musculoskeletal health. It is hypothesised vitamin D supplementation can improve musculoskeletal function and thereby reduce the incidence of falls. Vitamin D supplementation may be particularly beneficial in RAC settings as older people in RAC are deficient in vitamin D globally. Vitamin D deficiency in older adults is commonly caused by age, poor diet, lack of sunlight, decreased skin integrity, compromised metabolism, and polypharmacy (Weng and Lane, 2007; Verhoven et al., 2012; Holick, 2004; Mortensen et al., 2022). The prevalence of vitamin D deficiency in RAC could be as low as 8 % or as high as 94 % in cohorts with low use of vitamin D (Feehan et al., 2023). This large range can be attributed to a dearth of observational studies in RAC settings (Feehan et al., 2023; Lam et al., 2018). The Australian Therapeutic Guidelines recommend a dosage of 1000–2000 international units (IU) daily for older adults with vitamin D deficiency (Australian Medicines Handbook, 2020). In recognition of the need for adequate Vitamin D intake some nations fortify foods with vitamin D, for example in Australia it is mandatory to fortify oil spreads such as margarine with vitamin D (Food Standards Australia New Zealand, n.d.).

Universal vitamin D supplementation in RAC is recommended in the Australian Commission on Safety and Quality in Healthcare best practice guidelines for falls prevention in RAC (Australian Commission on Safety and Quality in Health Care, 2009) and by consensus guidelines (Gustavo et al., 2010). Recommendations applied to RACs are frequently derived from the randomised control trials conducted in community settings as evidence is limited in RAC settings; a Cochrane Review found moderate quality to support the use of vitamin D in falls based on four randomised control trials (Cameron et al., 2018). While there is some controversy on the impact of vitamin D supplementation and falls in community-settings, multiple large, high-quality trials have demonstrated a reduction in falls rates (Sanders et al., 2010; Glendenning et al., 2012; Khaw et al., 2017; Levis and Gómez-Marín, 2017; Hin et al., 2017) and that vitamin D supplementation is cost-effective due to the reduction in falls and associated costs (Thanapluetiwong et al., 2020). For example, a recent systematic review concluded that a vitamin D dosage of >800 IU a day reduced the rate of falls by approximately 5 % among older individuals in the community (Thanapluetiwong et al., 2020). Population-based studies from the United Kingdom (UK) and United States of America (USA) have demonstrated the cost-effectiveness of vitamin D supplementation for preventing falls in community settings (Poole et al., 2015; Lee et al., 2013). In 2014, providing universal vitamin D to people over 60 in the UK community was projected to save £420 million due to a decrease in the incidence of falls (Poole et al., 2015).

Research in RAC settings on the effects of vitamin D on falls reported inconclusive findings, partly due to study design limitations (Khaw et al., 2017; Levis and Gómez-Marín, 2017; Walker et al., 2017; Cameron, 2017; Walker et al., 2020). Study limitations of past research include inconsistencies in prescribing vitamin D supplements, low adherence to vitamin D intervention, high uptake of vitamin D in control groups, and a failure to consider certain subgroups of the population that may inherently have different risk levels (Feehan et al., 2023; Feehan et al., 2022; Cameron et al., 2018; Walker et al., 2020). To address the limitations of previous research we conducted a longitudinal cohort study using routinely collected data from 27 Sydney-based aged care facilities from one large Australian aged care provider. Our aims were to: 1) determine factors influencing the optimal adherence to vitamin D supplement usage, and 2) assess the association between use of vitamin D supplements and falls incidents over 36 months.

## 2 Methodology

### 2.1 Setting and design

We conducted a retrospective longitudinal cohort study using electronic data extracted from 27 RAC facilities within Sydney metropolitan areas in the state of New South Wales, Australia. The facilities were managed by a single aged care provider. The study period was from 1 July 2014–31 August 2022. This study is part of a broader research program investigating the application of predictive analytics and decision support for falls prevention in aged care (Ludlow et al., 2021). This study was reviewed and approved by the Macquarie University Human Research Ethics Committee (ID: 6144). This paper was structured in accordance with the REporting of studies Conducted using Observational Routinely-collected health Data (RECORD) statement (Benchimol et al., 2015).

### 2.2 Participants

Study inclusion criteria were residents who: were ≥65 years; had their first-time permanent admission into a RAC facility (with or without prior respite admission) on or after 1 July 2014; and those with >30 days of stay in the facility. Residents already residing in the RAC facilities before 1 July 2014 were not included in the study, as the focus was to explore the longitudinal use of vitamin D supplements and its relationship with falls starting from the time of facility entry. Temporary residents receiving respite care without subsequent permanent entry into RAC facilities were excluded from the study.

### 2.3 Data source

We obtained de-identified electronic data from the aged care provider. Similar datasets have been employed in previous studies (Wabe et al., 2022a; Wabe et al., 2022b; Huang et al., 2023). The datasets contained residents' demographics (e.g., age, gender), health conditions at the time of entry into RAC facilities (e.g., dementia status, osteoporosis), falls incidents (e.g., type of incident, date and time of incident, body region injured), and daily medication administration details (e.g., drug names and administration times).

Health conditions were recorded in a free-text field and we used a health macro that was previously developed by our research team (Lind et al., 2020), using similar electronic data, to identify relevant health conditions. We employed the World Health Organization's Anatomical Therapeutic Classification (ATC) codes to identify each distinct medication administered. For instance, we used ATC A11CC to identify vitamin D supplement usage, A12A for calcium and A11A or A11B for Multivitamins. The combination of vitamin A and D (ATC A11CB) was not used by residents in our cohort and thus not reported in this study.

### 2.4 Vitamin D use

Vitamin D usage status was assessed daily, starting from the time of admission, and continued for a maximum of three years. Given that most residents (>95 %) in our cohort were using a similar dose of 1000 IU, we chose to assess vitamin D usage as a binary indicator (yes/no), without considering the specific type of supplements, the frequency of administration, route, or dosage given. Vitamin D dosage was assessed using Australian prescribing guidelines which recommend a daily 800-1000 IU of vitamin D (Joshi et al., 2010).

**Fig. 1:**
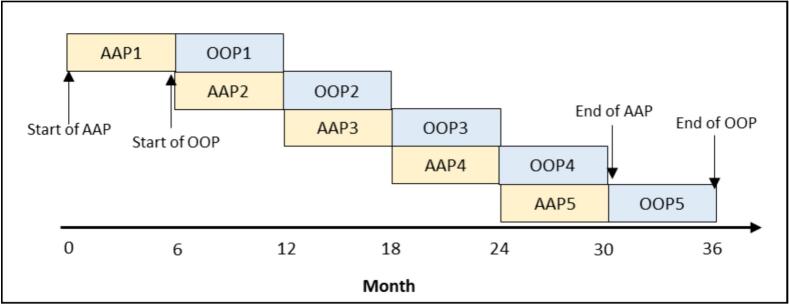
Implementation of vitamin D adherence assessment and outcome observation periods. AAP Vitamin D Adherence Assessment Period; OOP Outcome-Observation Period.

We used the Proportion of Days Covered (PDC) to assess the level of exposure or adherence to vitamin D use throughout the follow-up period. PDC represents the proportion of days that residents were covered by vitamin D supplements. For example, if a resident had a length of stay (LOS) of 130 days and received vitamin D supplements for 85 of those days, their PDC would be calculated as 65.4 %. The PDC metric is commonly used in pharmacoepidemiological studies to measure medication adherence (Choudhry et al., 2009; Wabe et al., 2017). The proportion of days covered by vitamin D supplements (*hereafter* PDC-vitamin D) was captured in a rolling time-varying manner every 6 months throughout the three-year study period (Fig. 1).

### 2.5 Outcome variable

The primary outcome measures were the number of *all falls* and *injurious falls* experienced by each resident. *All falls* was defined as occurrences of falls, regardless of whether they resulted in any bodily injury or necessitated hospital transfer. *Injurious falls* were defined as falls that resulted in some form of bodily injury, such as head injury. The secondary outcome measure was whether they achieved optimal adherence to vitamin D. The PDC approach was employed to determine the optimal adherence to vitamin D, with a value ≥80 % considered as the threshold for optimal adherence (Choudhry et al., 2009). The primary outcome measures were expressed as counts, while the secondary outcome measures were presented as binary outcomes.

### 2.6 Covariates

Several time-varying and time-invariant covariates were considered in the study. The *time*-*varying* covariates included: PDC-psychotropics (i.e., the proportion of days covered by at least one psychotropic medication (ATC N05 or N06)); PDC‑calcium (ATC A12A); PDC-analgesics (ATC N02); and PDC-CVD FRIDs (i.e., the proportion of days covered by at least one cardiovascular disease-based *falls risk increasing drugs*). CVD FRIDs included in the current study were diuretics (ATC C03), beta-blockers (ATC C07), calcium channel blockers (ATC C08) and renin-angiotensin-system (RAS) acting agents (ATC C09). Psychotropics, analgesics, and CVD FRIDs have been previously reported to be associated with an increased risk of falls (Seppala et al., 2018a; de Vries et al., 2018; Seppala et al., 2018b). The time-varying covariates were updated every 6 months, following a similar approach used for PDC-vitamin D. The *time*-*invariant* covariates consisted of age, gender, relevant health conditions (e.g., fracture history) based on a review of the literature and their relevance to the study, history of previous respite admissions into RAC, polypharmacy (defined as the concurrent use of 9 or more medications, excluding pro re nata ([PRN]/as needed medication)), year of admission to a RAC, and LOS.

### 2.7 Statistical analysis

We report descriptive statistics such as medians with inter-quartile ranges (IQR) as appropriate. We utilised a Kruskal-Wallis test for continuous variables and a Chi-Square test for categorical variables to compare the distribution of baseline characteristics in Table 1. Baseline medication-related variables were assess based on medication use during the initial two weeks after admission to an RACF. To identify factors associated with optimal adherence (PDC ≥80 %) to vitamin D supplement usage (Aim 1), we used logistic regression models with clustered-robust standard errors to account for potential correlation of observations within facilities. The analyses were adjusted for relevant baseline covariates including age, gender, health conditions, polypharmacy, LOS, and year of admission.

**Table 1:** Baseline participant characteristics by vitamin D supplement usage status.

Variable, n (%) unless otherwise specified	Total (*N* = 4520)	Ever received vitamin D suppl	
		No (*N* = 1457)	Yes (N = 3063)
Female	2861 (63.3 %)	837 (57.5 %)	2024 (66.1 %)*
Age, median (IQR)	86.0 (81.0–91.0)	86.0 (81.0–91.0)	87.0 (81.0–91.0)
Age group			
65–74	410 (9.1)	136 (9.3 %)	274 (8.9)
75–84	1366 (30.2)	469 (32.2 %)	897 (29.3)
85–94	2324 (51.4)	707 (48.5)	1617 (52.8)
≥ 95	420 (9.3)	145 (10.0)	275 (9.0)
Prior respite admission	2579 (57.1 %)	817 (56.1 %)	1762 (57.5 %)
**Health status**			
Dementia	2216 (49.0 %)	685 (47.0 %)	1531 (50.0 %)
Parkinson's disease	378 (8.4 %)	104 (7.1 %)	274 (9.0)*
Depression, mood & affective disorders	1815 (40.2 %)	536 (36.8 %)	1279 (41.8 %)*
Anxiety & stress-related disorders	1305 (28.9 %)	358 (24.6 %)	947 (30.9 %)*
Cerebrovascular accident	1122 (24.8 %)	364 (25.0 %)	758 (24.7 %)
Diabetes	1153 (25.5 %)	361 (24.8 %)	792 (25.9 %)
Thyroid	515 (11.4 %)	155 (10.6 %)	360 (11.8 %)
Chronic respiratory	833 (18.4 %)	265 (18.2 %)	568 (18.5 %)
Cancer	1312 (29.0 %)	461 (31.6 %)	851 (27.8 %)*
PUD & GORD	1409 (31.2 %)	441 (30.3 %)	968 (31.6 %)
Renal disease	863 (19.1 %)	285 (19.6 %)	578 (18.9 %)
Arthritis	2491 (55.1 %)	788 (54.1 %)	1703 (55.6 %)
Osteoporosis	1234 (27.3 %)	350 (24.0 %)	884 (28.9 %)*
Fracture	1475 (32.6 %)	375 (25.7 %)	1100 (35.9 %)*
Falls history*	2274 (50.3 %)	670 (46.0 %)	1604 (52.4 %)*
**Medication** (during the first 2 weeks after admission to RAC)			
Total no. of medications, median (IQR)	8.0 (5.0–11.0)	7.0 (4.0–10.0)	8.0 (5.0–11.0)*
Polypharmacy without PRN	2020 (44.7 %)	536 (36.8 %)	1484 (48.4 %)*
*ATC classification level 1 of medications used*			
Alimentary tract and metabolism	3739 (82.7 %)	1098 (75.4 %)	2641 (86.2 %)*
Anti-infectives for systemic use	1282 (28.4 %)	394 (27.0 %)	888 (29.0 %)
Blood and blood forming organs	2526 (55.9 %)	737 (50.6 %)	1789 (58.4 %)*
Cardiovascular system	3264 (72.2 %)	1019 (69.9 %)	2245 (73.3 %)*
Dermatologicals	828 (18.3 %)	262 (18.0 %)	566 (18.5 %)
Genito urinary system and sex hormones	614 (13.6 %)	195 (13.4 %)	419 (13.7 %)
Musculo-skeletal system	790 (17.5 %)	261 (17.9 %)	529 (17.3 %)
Nervous system	3339 (73.9 %)	1050 (72.1 %)	2289 (74.7 %)
Respiratory system	892 (19.7 %)	282 (19.4 %)	610 (19.9 %)
Sensory organs	1220 (27.0 %)	391 (26.8 %)	829 (27.1 %)
Systemic hormonal preparations	943 (20.9 %)	308 (21.1 %)	635 (20.7 %)
*Use of Falls*-*risk increasing drugs* (*FRIDs*)			
Psychotropic medications	2184 (48.3 %)	684 (46.9 %)	1500 (49.0 %)
Antipsychotics	546 (12.1 %)	192 (13.2 %)	354 (11.6 %)
Anxiolytics/sedatives/hypnotics	442 (9.8 %)	148 (10.2 %)	294 (9.6 %)
Antidepressants	1338 (29.6 %)	405 (27.8 %)	933 (30.5 %)
Antiepileptics	657 (14.5 %)	199 (13.7 %)	458 (15.0 %)
Analgesics (opioid or NSAIDs)	2454 (54.3 %)	769 (52.8 %)	1685 (55.0 %)
Any Cardiovascular FRIDs	2899 (64.1 %)	906 (62.2 %)	1993 (65.1 %)
Diuretics	1134 (25.1 %)	364 (25.0 %)	770 (25.1 %)
Beta-blockers	1266 (28.0 %)	388 (26.6 %)	878 (28.7 %)
Calcium channel blockers	769 (17.0 %)	233 (16.0 %)	536 (17.5 %)
RAS acting agents	1401 (31.0 %)	420 (28.8 %)	981 (32.0 %)*
*Supplements*			
Any vitamins including vitamin D	2376 (52.6 %)	203 (13.9 %)	2173 (70.9 %)*
Calcium supplement	530 (11.7 %)	178 (12.2 %)	352 (11.5 %)
Multivitamins	512 (11.3 %)	131 (9.0 %)	381 (12.4 %)*
Year of admission*			
2014	214 (4.7 %)	68 (4.7 %)	146 (4.8 %)
2015	465 (10.3 %)	129 (8.9 %)	336 (11.0 %)
2016	501 (11.1 %)	158 (10.8 %)	343 (11.2 %)
2017	641 (14.2 %)	194 (13.3 %)	447 (14.6 %)
2018	592 (13.1 %)	187 (12.8 %)	405 (13.2 %)
2019	570 (12.6 %)	184 (12.6 %)	386 (12.6 %)
2020	451 (10.0 %)	138 (9.5 %)	313 (10.2 %)
2021	664 (14.7 %)	219 (15.0 %)	445 (14.5 %)
2022	422 (9.3 %)	180 (12.4 %)	242 (7.9 %)

PUD/GORD, Peptic Ulcer Disease/Gastro-Oesophageal Reflux Disease; ATC, Anatomical Therapeutic Chemical; RAS, Renin-angiotensin-system. *P < 0.05.

To assess the longitudinal association between vitamin D use and subsequent falls we implemented a rolling time-varying *predictor*-*outcome* approach. The main predictor variable of interest was vitamin D supplement use, measured by PDC-vitamin D, and the outcome measure was the number of falls. As illustrated in Fig. 1, the study data comprised five pairs of vitamin D adherence assessment periods (AAP) and outcome observation periods (OOP). Each resident could have up to 5 sets of data, one for each adherence-outcome period pair. The vitamin D adherence level (PDC-vitamin D) measured during AAP1 was utilised to predict falls incidents in OOP1, and this process was repeated sequentially until the end of the study period. During each AAP, residents who did not have a complete subsequent OOP were excluded from the analysis for that specific period. For example, if an individual had a LOS of 14 months, they would be included in the first adherence-outcome pair but not in the second one, even if they completed the second Vitamin D adherence period. This exclusion ensures that the analysis only includes residents who have sufficient data for both the adherence and outcome periods, allowing for a more reliable examination of the relationship between vitamin D adherence and falls within each specific time frame. The rolling predictor-outcome approach offers an advantage over the traditional concurrent assessment of predictor and outcome variables, as it reflects potential cause-effect relationships.

Subsequently, we applied a Generalized Estimating Equations (GEE) to model the longitudinal association between PDC-vitamin D and number of falls while adjusting for confounders. We used robust standard errors to accommodate the panel nature of the data, which involved multiple repeated measures of observation for residents, a negative binomial distribution with a log link function to handle overdispersion of the count outcome and unstructured working correlation matrix to allow for a flexible and unconstrained estimation of the correlations between measurements. In the GEE model, we computed the Incident Rate Ratio (IRR) along with 95 % Confidence Intervals (CIs) after adjusting for the baseline covariates mentioned earlier, as well as the following time-varying covariates: PDC-psychotropics, PDC‑calcium, PDC-Analgesics, and PDC-CVD FRIDs. To aid interpretation, all the PDC measures were reported as every 10 % increase. Further, we introduced the lagged outcome as a time-varying confounder variable in the GEE model to account for the potential influence of past falls on future occurrences. This involved using the number of falls that occurred in the past 6-month interval as one of the covariates to predict the number of falls that might occur in the next 6-month interval. The inclusion of this lagged variable is crucial as a history of falls is a powerful predictor of future falls (Vaishya and Vaish, 2020).

We also conducted sub-group analyses stratified by gender and selected baseline status including gender, osteoporosis/fracture, and dementia to assess the robustness of our primary findings. All *P*-values were two-tailed, and statistical significance was set at *P* < 0.05. The analysis was carried out using Stata version 18 (StataCorp LP, College Station, TX).

## 3 Results

### 3.1 Participants

**Fig. 2:**
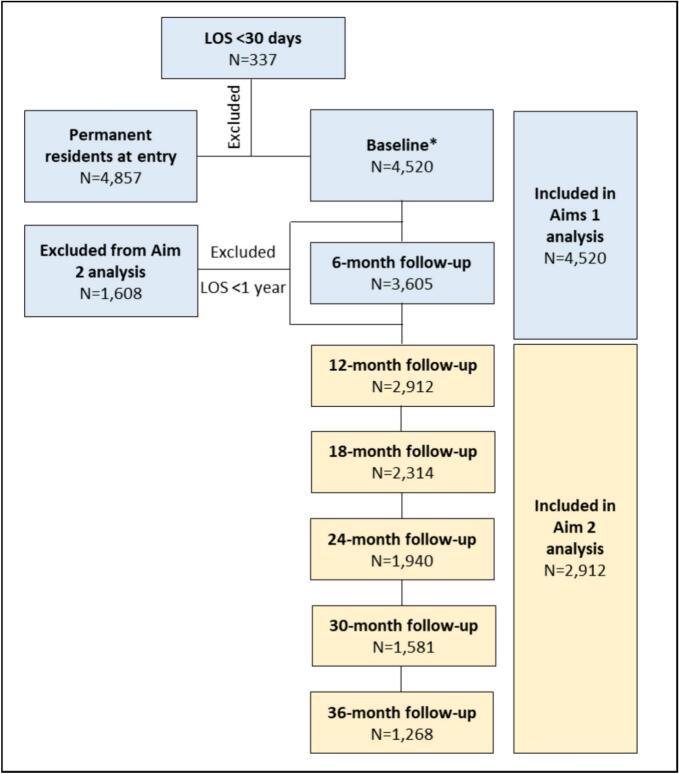
Participants selection flowchart. LOS, Length of stay. *We used the first two weeks of entry to determine baseline medication usage for eligible participants.

The study sample consisted of 4520 residents, 63.3 % were female and the median age was 86 years. Table 1 presents the baseline characteristics by vitamin D supplement usage status. Although most characteristics were comparable between the two groups, some differences were observed. At entry into the RAC facility, 43.9 % of residents (*n* = 1983) were using vitamin D supplements. However, over a 36 month follow up period in the RAC facility 67.8 % (*n* = 3063) received vitamin D at least once during their stay in RAC facilities. Over half of the residents (*n* = 2376) were using at least one type of vitamin (including vitamin D) at baseline, and 11.3 % (*n* = 520) were using calcium supplements (Table 1). In total 2912 residents (64.4 %) were included at the 12-month follow-up (Fig. 2).

### 3.2 What factors influence optimal adherence to vitamin D supplement usage?

Among residents who used vitamin D supplements (*n* = 3063), the median PDC-vitamin D was 74.8 % (IQR 36.3–91.6) indicating that about half of all residents received daily vitamin D supplements for 75 % of their total time spent in the RAC facilities. The proportion of residents who achieved an optimal adherence (i.e., PDC-vitamin D ≥ 80 %) was 44.6 % (*n* = 1365). Increasing age, a history of osteoporosis or fracture, and having dementia were associated with a greater likelihood of achieving optimal adherence. Conversely, the presence of diabetes, anxiety and stress-related disorders, or cancer was linked to a reduced likelihood of attaining optimal adherence. For instance, residents with a history of osteoporosis or fracture had a 23 % greater likelihood of achieving optimal adherence (OR 1.23; 95 % CI 1.06–1.42; *P* = 0.005), and those with diabetes had a 31 % reduced likelihood of achieving optimal adherence (OR 0.69; 95 % CI 0.56–0.84; *P* < 0.001) compared to their counterparts after adjusting for confounders (Table 2).

**Table 2:** Factors associated with an optimal adherence to vitamin D supplement usage (PDC-vitamin D ≥ 80 %).

Variable	PDC-vitamin D ≥ 80 % (*n* = 3063)			
	OR	95 % CI of OR		P
		Lower	Upper	
Female vs male	1.12	0.93	1.36	0.222
Age group [Ref = 65–74]				
75–84	1.38	1.00	1.92	0.052
85–94	1.61	1.23	2.12	0.001
≥95	2.00	1.37	2.94	0.000
Prior respite admission	1.04	0.85	1.26	0.715
Health status [Ref = No disease]				
Dementia	1.33	1.14	1.55	0.000
Parkinson's disease	0.93	0.70	1.24	0.635
Depression, mood & affective disorders	1.02	0.85	1.22	0.817
Anxiety & stress-related disorders	0.85	0.73	0.99	0.035
Cerebrovascular accident	1.10	0.99	1.23	0.079
Diabetes	0.69	0.56	0.84	0.000
Thyroid	0.93	0.72	1.19	0.544
Chronic respiratory	0.85	0.68	1.05	0.137
Cancer	0.77	0.65	0.92	0.003
PUD & GORD	0.90	0.78	1.03	0.130
Renal disease	0.98	0.83	1.16	0.833
Arthritis	0.98	0.84	1.13	0.733
Osteoporosis/fracture	1.23	1.07	1.42	0.004
Falls history	1.15	0.99	1.34	0.069
Polypharmacy	0.90	0.76	1.07	0.243
Length of stay in years (Ref ≤ 1 year)				
1–2	1.13	0.89	1.44	0.321
2–3	0.96	0.69	1.35	0.836
3–4	0.89	0.69	1.14	0.357
≥5	0.70	0.47	1.05	0.082
Year of admission [Ref = 2014]				
2015	1.50	0.96	2.35	0.076
2016	1.02	0.58	1.82	0.936
2017	1.24	0.75	2.04	0.403
2018	1.45	0.79	2.64	0.231
2019	1.02	0.55	1.89	0.960
2020	1.27	0.72	2.25	0.408
2021	2.11	1.13	3.95	0.020
2022	2.31	1.34	3.99	0.003

**Fig. 3:**
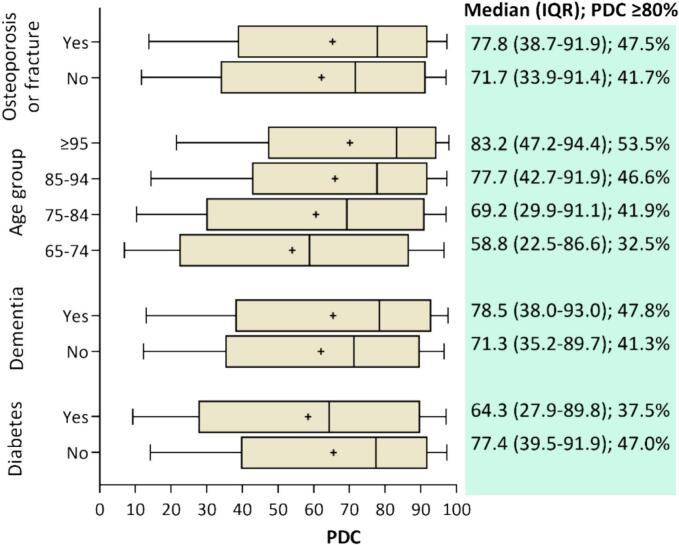
Boxplots and proportion of residents achieving optimal adherence (PDC-vitamin D ≥ 80 %) for selected variables. Boxes represent the IQR (25th and 75th percentiles) with the median value within the boxes, the mean value is represented as a ‘+’ and the capped bars represent the 10th and 90th percentiles.

Fig. 3 highlights differences in median PDC-vitamin D and optimal adherence proportions across key variables. For example, residents aged ≥95 years exhibited a median PDC-vitamin D of 83.2 % with 53.2 % achieving optimal adherence. In comparison, those aged 65–74 years had a median PDC-vitamin D of 58.8 % with 32.5 % achieving optimal adherence.

### 3.3 Association between vitamin D use and number of falls

Over three-quarters of residents (75.9 %, *n* = 3429) experienced a fall and 60.5 % (*n* = 2734) of residents experienced fall causing injury. In total there were 28,280 falls recorded during 3,511,497 resident days, resulting in a crude fall incident rate of 8.05 per 1000 resident days (IQR 7.96–8.15). Of all falls, 36.3 % (*n* = 10,267) were injurious falls; 2.92 per 1000 resident days (2.87–2.98).

Table 3 presents the results of multivariate GEE models. In analyses that incorporated a diverse range of covariates—both time-varying and time-invariant—into the model, no statistically significant associations were observed between the use of vitamin D supplements (as measured by PDC-vitamin D) and the number of falls. This absence of associations held true for both study outcomes: all falls (IRR 1.01; 95 % CI 1.00–1.02; *P* = 0.237) and injurious falls (IRR 1.01; 95 % CI 1.00–1.02; *P* = 0.091).

**Table 3:** GEE model showing predictors of *all falls* and *injurious falls* (*n* = 2912).

Variable	All falls				Injurious falls			
	IRR	95 % CI of IRR		P	IRR	95 % CI of IRR		P
		Lower	Upper			Lower	Upper	
***Time***-***varying***								
PDC-vitamin D [every 10 % increase]	1.01	1.00	1.02	0.229	1.01	1.00	1.02	0.092
PDC‑calcium [every 10 % increase]	1.01	1.00	1.03	0.041	1.01	1.00	1.03	0.115
PDC-psychotropics [every 10 % increase]	1.02	1.01	1.03	0.000	1.02	1.00	1.03	0.013
PDC-analgesics [every 10 % increase]	1.01	1.00	1.02	0.043	1.01	1.00	1.02	0.032
PDC-CVD FRIDs [every 10 % increase]	1.00	0.99	1.01	0.643	0.99	0.98	1.00	0.049
Number of falls in last 6 months	1.13	1.12	1.15	0.000	1.12	1.11	1.13	0.000
***Time***-***invariant***								
Polypharmacy	0.93	0.85	1.03	0.160	1.04	0.93	1.17	0.480
Male vs female	1.50	1.36	1.65	0.000	1.68	1.51	1.87	0.000
Age group [Ref = 65–74]								
75–84	1.21	1.01	1.44	0.035	1.42	1.17	1.73	0.000
85–94	1.37	1.16	1.63	0.000	1.94	1.60	2.34	0.000
≥95	1.51	1.22	1.87	0.000	2.34	1.83	3.00	0.000
Prior respite admission [Ref = No]	1.11	1.01	1.21	0.023	1.14	1.03	1.26	0.009
Health status [Ref = No disease]								
Dementia	1.53	1.40	1.68	0.000	1.43	1.29	1.60	0.000
Parkinson's disease	1.42	1.21	1.66	0.000	1.32	1.12	1.56	0.001
Depression, mood & affective disorders	1.04	0.95	1.15	0.389	1.12	1.00	1.25	0.042
Anxiety & stress-related disorders	1.04	0.95	1.14	0.380	1.07	0.96	1.19	0.208
Cerebrovascular accident	1.07	0.97	1.18	0.155	1.04	0.94	1.17	0.437
Diabetes	0.99	0.89	1.10	0.796	0.98	0.86	1.11	0.744
Thyroid	1.00	0.88	1.15	0.952	1.03	0.89	1.20	0.664
Chronic respiratory	1.01	0.90	1.13	0.891	1.02	0.90	1.17	0.714
Cancer	1.00	0.91	1.10	0.975	1.06	0.95	1.18	0.295
PUD & GORD	0.99	0.90	1.08	0.820	0.92	0.83	1.02	0.113
Renal disease	0.96	0.86	1.08	0.498	0.90	0.79	1.03	0.120
Arthritis	1.00	0.91	1.09	0.980	0.97	0.88	1.07	0.558
Osteoporosis/fracture	1.12	1.02	1.22	0.016	1.20	1.08	1.33	0.001
Falls history	1.21	1.10	1.32	0.000	1.25	1.13	1.38	0.000
Year of admission [Ref = 2014]								
2015	0.86	0.69	1.07	0.178	0.95	0.74	1.22	0.688
2016	1.06	0.86	1.30	0.611	1.03	0.80	1.31	0.833
2017	0.97	0.79	1.20	0.797	0.97	0.76	1.23	0.802
2018	0.95	0.78	1.17	0.641	1.03	0.81	1.30	0.836
2019	1.21	0.98	1.50	0.080	1.29	1.01	1.64	0.043
2020	0.92	0.74	1.14	0.429	0.93	0.72	1.20	0.559
2021	1.16	0.92	1.47	0.209	1.06	0.79	1.42	0.700

Subgroup analyses based on gender and the presence of a history of osteoporosis/fracture showed no notable distinctions between the respective groups. We found no association between the use of vitamin D supplements and the number of all falls for both males (*P* = 0.216) and females (*P* = 0.428); or for those individuals with a baseline history of osteoporosis/fracture (*P* = 0.311) or not (*P* = 0.540). Nonetheless, a sub-group analysis by dementia status revealed that for residents with dementia, an increased use of vitamin D supplements was associated with a small, yet statistically significant increase in the rate of both all falls (IRR 1.01; 95 % CI 1.00–1.03; *P* = 0.025) and injurious falls (IRR 1.02; 95 % CI 1.00–1.03; *P* = 0.014). While the statistical significance of a slight increase in the falls rate is evident, its clinical significance may be negligible. This association was not evident among residents without dementia (Table S1).

## 4 Discussion

In our study, 44 % of residents achieved optimal adherence to Vitamin D with adherence greater in older, female residents compared to younger male residents. We found no association between vitamin D use, with or without calcium supplementation, and the incidence of falls. In subgroup analyses, residents with a history of osteoporosis showed no decrease in falls risk with increased adherence to vitamin D supplementation. Conversely, residents with dementia experienced increases in both all falls and injurious falls with increasing adherence to vitamin D supplementation. However, while this increase was statistically significant, it is unlikely to be clinically significant.

Evidence from our cohort analysis suggests that 1000 IU of vitamin D with or without calcium is not effective for falls prevention in RAC. Our findings are consistent with some other literature, including randomised control trials (RCTs) and meta-analyses, which have found no improvement in the incidence of falls with vitamin D supplementation with or without calcium supplementation in RAC (Banerjee et al., 2021; Bolland et al., 2014). Studies and interventions of vitamin D supplementation in the general population have also found similar results of vitamin D supplementation with no improvement in falls (Barr et al., 2010; LeBoff et al., 2020). Together this evidence suggests that vitamin D as a single intervention is not sufficient to impact the incidence of falls in a population that has a very high risk of falling. However, this is contrary to some other high-quality evidence; a Cochrane Review conducted in 2018 which found moderate-quality evidence that vitamin D reduces the rate of falls by 7 % across four studies in RAC (Cameron et al., 2018). The discrepancy may be due to the low number and small nature of studies historically conducted in this setting. As more research is conducted in RAC, it is important that high quality reviews continue to be updated to inform best practice.

Our study adds new evidence for people in RAC. In a subgroup analysis we found residents who had a history of osteoporosis (used as a proxy for vitamin D deficiency as the rate of deficiency remains high in this population despite diagnosis and treatment with vitamin D) (Zhu et al., 2023) did not experience a reduction in the incidence of falls with vitamin D supplementation. This is contrary to some studies in community settings have found that vitamin D supplementation was more likely to reduce the incidence of falls in those who were deficient in vitamin D at baseline (Murad et al., 2011; Guo et al., 2014; Ling et al., 2021). However, the observation in our cohort study may be caused by the proxy use of osteoporosis rather than true vitamin D deficiency. Additionally, in our study we found that falls increased in people with dementia receiving vitamin D. While this is likely not clinically significant it is important to consider the cause of this potential increase in risk. There is evidence indicating that vitamin D supplementation could improve cognitive function and play a potential role in preventing the progression of dementia (Harse et al., 2023; Ghahremani et al., 2023). It is possible that the impact on cognition impacts function and ultimately falls risks in residents. However, further research is needed to thoroughly investigate and confirm the impact of vitamin D use, including the effects of different dosage strengths, in this population within RAC.

It is possible that vitamin D supplementation is effective at reducing the risk of falls in RAC, but this study was not able to detect this effect due to inconsistent use of vitamin D across our study population. This is evident as baseline where older, female long-term residents with a history of falls and osteoporosis were the most likely population subgroup to adhere to vitamin D use. This is consistent with international research (Walker et al., 2020; Yanamadala et al., 2012; Hamid et al., 2007; Kennedy et al., 2015; Nordic Council of Ministers, 2014), but could have skewed results. It was also evident throughout resident stay; the group adhering to vitamin D use was predominantly composed of older women with osteoporosis. However, the role of this bias in the interpretation of our results is double-edged; the population most adherent to vitamin D use in RAC are also, theoretically, the most likely to show an improvement in falls incidence if vitamin D is effective at preventing falls (Ling et al., 2021). Additionally, but not a fault of our study design, while dosages prescribed often followed current prescribing guidelines there is limited international evidence to suggest that a dosage of 1000 IU is sufficient for aged care residents to achieve the optimal range of serum vitamin D (50nMol/L).

### 4.1 Implications for policy and practice

Our study suggests that vitamin D supplementation with or without calcium supplementation does not impact the rate of falls in RAC settings. The results of our study should not alter the importance of addressing vitamin D deficiency in RAC from a nutrition standpoint. Afterall, vitamin D impacts many organs in the body including heart, bone, and muscle function. Notably, vitamin D use may still reduce the incidence of fall related fractures in this population; this fall-related outcome is understudied in RAC and exploring this association was not possible in our dataset (Banerjee et al., 2021). However, the results do serve to moderate the expectations and effectiveness as vitamin D as a falls prevention intervention in RAC and its recommendation in future clinical guidelines for falls prevention purposes.

### 4.2 Strengths and limitations

Key strengths of our study are the size of the RAC dataset, including the time period, number of residents and facilities contained within it. This quantity of data enabled sub-group analyses which have been infrequently conducted in RCTs. Our methods are another strength, we used a time-varying predictor outcome approach. However, this did lead to the loss of some participants. Indeed, previous intervention studies have faced difficulties due to the increased use of vitamin D in control groups (Gustavo et al., 2010). The ethical and logistical hurdles of establishing a genuine control group in interventional studies are noteworthy, especially since numerous residents in RAC facilities have vitamin D deficiency and require supplementation. Concurrently, there is a growing trend in routine care to align with the recommendations in the Australian Therapeutic Guidelines for addressing vitamin D deficiency in RAC. Therefore, longitudinal cohort studies may compliment future approaches to study vitamin D in RAC.

Our study has some limitations. In our analysis, we were not able to account for the functional status of residents as this information is not available in our dataset. This missing information could be a potential confounding factor in our analysis as it is possible that vitamin D is prescribed to frailer residents. In our analysis we also could not explore the effect of vitamin D or calcium dose on the incidence of falls and injurious falls. This is because residents received similar doses (∼1000 IU). While this is positive for the residents (as they received treatment consistent with prescribing guidelines, 1000 IU daily) (Australian Medicines Handbook, 2020) it limits our ability to explore the possible association between dose and falls. Another limitation of study was, we were not able to explore the association between vitamin D supplementation with or without calcium on the incidence of fall related fractures. There is limited evidence available on this relationship in RAC populations internationally and is an important area for future research to address.

## 5 Conclusion

Our findings suggest that vitamin D with or without calcium supplementation is not an effective falls prevention intervention in RAC. In practice, clinicians should ensure that residents meet adequate vitamin D intake to maintain their nutritional health. However, it should not be used as a standalone falls prevention intervention in RAC populations.

## Abbreviations

AAD: Adherence assessment periods
ATC: Anatomical Therapeutic Chemical
ATC: Anatomical Therapeutic Classification
CI: Confidence interval
CVD: Cardiovascular disease
FRID: Fall Risk Increasing Drugs
GEE: Generalized Estimating Equations
IQR: Inter quartile range
IRR: Incident rate ratio
IU: International Unit
LOS: Length of stay
OOP: Outcome observation periods
PDC: Proportion of days covered
PRN: Pro ne nata
PUD/GORD: Peptic Ulcer Disease/Gastro-Oesophageal Reflux Disease
RAC: Residential aged care
RAS: Renin-angiotensin-system
RCT: Randomised control trial
RECORD: REporting of studies Conducted using Observational Routinely-collected health Data
UK: United Kingdom
USA: United States of America

The following is the supplementary data related to this article.

Table S1 GEE model: sensitivity analysis by gender, a history of osteoporosis/fracture and dementia status. All the analyses were adjusted for variables in Table 3. Table S1

## CRediT authorship contribution statement

**Nasir Wabe:** Writing – review & editing, Writing – original draft, Visualization, Software, Methodology, Formal analysis, Conceptualization. **Isabelle Meulenbroeks:** Writing – review & editing, Writing – original draft, Conceptualization. **Desiree C. Firempong:** Writing – review & editing, Writing – original draft. **Magdalena Z. Raban:** Writing – review & editing, Supervision. **Amy D. Nguyen:** Writing – review & editing, Project administration. **Jacqueline T. Close:** Writing – review & editing, Supervision. **Stephen R. Lord:** Writing – review & editing, Supervision. **Johanna I. Westbrook:** Writing – review & editing, Supervision, Funding acquisition.

## Declaration of competing interest

The authors have no conflicts of interest to declare.

## Data Availability

The data that has been used is confidential.
