# Supplementary material for: Vitamin D supplementation and falls in residential aged care: A longitudinal multisite cohort study
Source: Bone Rep. 2024 Jul 23;22:101791. doi: 10.1016/j.bonr.2024.101791 (PMC11321375; doi:10.1016/j.bonr.2024.101791)
Supplement: Table S1 — GEE model: sensitivity analysis by gender, a history of osteoporosis/fracture and dementia status. All the analyses were adjusted for variables in Table 3. [file mmc1.docx]

**Supplementary File**

**Table S1: GEE model: Sensitivity analysis by gender, a history of osteoporosis/fracture and dementia status**. All the analyses were adjusted for variables in Table 3.

| PDC-vitamin D [every 10% increase] | | **All falls** | | | | **Injurious falls** | | | |
| --- | --- | --- | --- | --- | --- | --- | --- | --- | --- |
|  |  | IRR | 95% CI of OR | | P | IRR | 95% CI of OR | | P |
|  |  |  | Lower | Upper |  |  | Lower | Upper |  |
| Gender | Male | 1.01 | 0.99 | 1.03 | 0.221 | 1.01 | 0.99 | 1.03 | 0.208 |
|  | Female | 1.00 | 0.99 | 1.02 | 0.426 | 1.01 | 1.00 | 1.02 | 0.185 |
| Osteoporosis/fracture | Yes | 1.01 | 0.99 | 1.02 | 0.340 | 1.01 | 0.99 | 1.03 | 0.315 |
|  | No | 1.00 | 0.99 | 1.02 | 0.518 | 1.01 | 0.99 | 1.03 | 0.206 |
| Dementia | Yes | 1.01 | 1.00 | 1.03 | 0.022 | 1.02 | 1.00 | 1.03 | 0.013 |
|  | No | 1.00 | 0.98 | 1.02 | 0.885 | 1.00 | 0.98 | 1.02 | 0.834 |
